# Automation, regulation, and collaboration: Threats and opportunities for clinical medical physics careers in diagnostic imaging and nuclear medicine

**DOI:** 10.1002/acm2.12604

**Published:** 2019-05-13

**Authors:** David W. Jordan, Eric L. Gingold, Ehsan Samei

**Affiliations:** ^1^ Department of Radiology University Hospitals Cleveland Medical Center & Case Western Reserve University Cleveland OH USA; ^2^ Department of Radiology Thomas Jefferson University Philadelphia PA USA; ^3^ Department of Radiology Duke University Medical Center Durham NC USA

Being a medical imaging physicist seems fraught with existential anxiety these days. Uncertainties abound. To respond, we need to change, but we need to protect the gains we have made as a profession as well. Considering our progressive track record, the future of the profession ought to be bright, but there are threats all around us that we cannot underestimate. We have a unique skill set and value proposition and there are too few of us to sustain the healthcare enterprise, yet there are too many un‐ or under‐qualified individuals encroaching into our scope of practice; meanwhile, concerns abound that our jobs will be subsumed by robots and artificial intelligence. We seem to need to do more of everything — research, teaching, quality improvement, developing leadership skills — but how can we be careful not to do too much and burn out? We need to eliminate distractions so that we can engage in thoughtful work, but we need to be available and responsive in order to support clinicians, especially regarding safety.

Thus, it seems daunting to decide on the first priority each day, let alone to ponder the future of our profession.

Nevertheless, in the midst of uncertainty and change, we should consider our own future and how it fits into that of our larger healthcare environment. Recently, an editorial in this journal discussed the state of the medical physics profession in 2019 in terms of *culture*, quoting extensively from “the HP Way.”^1^ This was not a discussion about accreditation, radiation output calibrations, creation of databases using informatics tools, or other routine medical physics matters; rather, it drew on a set of affirmations about behaviors, expectations, and intentions, speaking to the way an organization will be and how it will do its work.

Culture can arise spontaneously or be created deliberately. To secure the future of medical imaging physics, we urge each individual medical physicist, and each practice group, to develop and articulate clear statements of mission, vision, and value proposition through deliberate strategic planning. These elements together shape the culture of practices and the profession as a whole. The HP Way is a comprehensive statement that incorporates all of these elements and specific expectations about how HP employees fulfill them.

A *mission statement* describes action and intention, and *vision statements* describe ideal states. An effective vision statement allows examination of gaps between current and ideal conditions. *Value propositions* follow from mission and vision. If an organization acts in full accordance with its mission, and if its vision is realized, who benefits, and how? Put another way: to whom do the mission and vision matter, and why? An “elevator speech” is a short, conversational expression of a value proposition, and can be composed by answering these questions.

AAPM's Medical Physics 3.0 initiative is a blueprint for culture within AAPM and the medical physics profession,^2^ describing a vision wherein medical physics improves the health of every patient. To accomplish this, medical physicists apply the science of physics to medicine, functioning as both scientists *and* healthcare providers. Medical Physics 3.0 provides elements of mission, vision, and value proposition that medical physicists should consider for their own practices.

We propose missions and visions for medical physics practice that incorporate rigorous scientific thought and action, service orientation toward patients and other healthcare professionals, and systems‐based execution of our roles in healthcare. In these contexts, we should be mindful of opportunities to provide competent leadership (in formulating our value propositions) and to raise awareness of how medical physics contributes to patient health (in communicating our value propositions).

We can translate our planning into action by considering three key questions:

“What should we *start* doing?”
“What should we *stop* doing?”
“What should we *keep* doing?”

The answers will vary according to each practice's strategic plan, which should include an analysis of its strengths, weaknesses, opportunities, and threats.

AAPM Report 301^3^ identifies consultative, project, and development work (Level 2 and Level 3 services) as growth opportunities for imaging physicists. Indeed, the market for expertise to solve complex problems seems likely to grow faster than the installed base of imaging equipment. These needs are growing for all healthcare providers; both consulting and in‐house physicists are fielding increasing requests for this kind of support. Level 2 encompasses services such as providing education; these are not mandatory as per regulations, but many institutions want or need them, and there can be a reasonable degree of standardization (e.g., using published curriculum guidelines). Level 3 services are more exploratory and less well‐standardized, such as a project to select, install, configure, and implement a software tool to detect artifacts in clinical images. AAPM Report 301 acknowledges that there may be minimal (or no) external motivation for facilities to undertake these efforts without accreditation or regulatory mandates. Therefore, growth will only occur if medical physicists have the skills to do the work and opportunities to demonstrate its value. As a profession, one might consider whether to drive this growth, we should: start more task groups to provide references and resources supporting this kind of work; stop spending so much time on hands‐on equipment testing; and keep engaging students, residents, and junior colleagues in projects to develop their skills.

Of course, a recommendation to spend less time testing equipment is fraught. Testing is mandatory and must meet specific standards to ensure quality, safety, and compliance. It is a major component of imaging physics work, representing a substantial portion of the practice for many consulting groups. Many of us enjoy this work and do it well. Aside from feeling threatened, such a proposal probably also seems impractical; we cannot abandon these duties, nor can we delegate them to others who are not qualified to do them in our place.

This dilemma should spur us to revisit the three questions. Should we start to train technologists, assistants, or trainees to perform some testing under appropriate supervision to reduce our time burden? Should we start to develop tools, documentation, and perhaps even technologies using artificial intelligence? In the short term, after these changes make equipment testing less time‐consuming, they will lessen the need for medical physicists to perform these functions personally. Does this threaten our job security? Seemingly, although our original objective was to liberate time and attention for Level 2 and Level 3 projects and services, which are less amenable to automation and outsourcing. By proactively automating and outsourcing the tasks we can, we position ourselves for a more secure future. While we do so, we should keep doing hands‐on work such as radiation measurements and operating imaging equipment. Our direct familiarity with the technology informs our ability to solve problems and positions us at the intersection between clinicians and engineers. We cannot be effective if these activities dominate our time and attention, but we will struggle to be effective if we do not do them at all.

For radiation therapy physicists, there is growing interest in direct patient contact and care.^4^, ^5^ There may or may not be a parallel “should” here for medical imaging physicists to have direct contact with patients for certain procedures. Regardless, we undoubtedly should increase the quantity and quality of our contact with *other healthcare professionals*. Physicians and radiation therapy physicists rely heavily on quality imaging and generally regard skilled imaging physicists as a precious resource. In these engagements with either patients or other healthcare professionals, medical imaging physicists in the consulting environment have both a challenge and an opportunity. The daily logistics of consulting make it difficult to have face‐to‐face contact with key clinical personnel, and physician contact can pose the greatest challenge. Consulting physicists may not be able to foster relationships via regular meetings or chance hallway or cafeteria encounters. Yet the nature of the consulting service model means that these physicists often excel in the domain of “soft skills” that create and sustain these relationships. While our potential impact depends on our technical acumen, fostering these relationships is an important exercise in leadership and improves the awareness of our profession and the value we provide.^6^, ^7^

While “leadership” is often taken to be synonymous with the skills and functions of supervisory, managerial, or executive roles, it has a broader meaning that applies to every practicing medical physicist. Leadership here refers to the skills and motivations that connect the medical physicist's knowledge and abilities to problem‐solving in the real world. In essence, leadership is the catalyst for delivering value. For some, this might mean serving as a supervisor, manager, chief, or director. For everyone, it means: cultivating the interpersonal skills to connect with someone who has a problem to solve; gathering information to understand the problem; reaching agreement on a path forward; and ultimately, arriving at a shared understanding of what has been accomplished. It also means the initiative to seek opportunities to share knowledge, apply skills, and solve problems.

The trend toward increasingly prescriptive regulatory and accreditation requirements in imaging and radiation protection is narrowing the perceived definition of the medical imaging physicist's role in healthcare delivery. Some conclude that such mandates improve our job security and prevent our displacement by automation or outsourcing. This trend could also be considered a threat: by driving the field toward “cookbook” approaches that are simpler to regulate, but also easier to automate or outsource to other workers, the rules establish the qualified medical physicist by fiat, but undermine the need for such individuals to actually do the work. At the end of this road, the only thing requiring a qualified medical physicist's involvement is the law or regulation. Hospital associations and other cost‐conscious stakeholders have enough influence to eliminate these job‐securing provisions, should they be so inclined, clearing the way for the work to be done by machines, technicians, assistants, and others. In the meantime, our involvement in these mandatory activities diverts our time and attention from other roles requiring our unique skills, such as innovation.

It would be unwise to presume a future in which current regulatory and accreditation rules are permanent; these can be changed or rescinded at any time and with little notice. Accordingly, we should not rely on them to create demand for our contributions. Compliance with regulations is often used as a proxy for safety and quality, but it is not the same thing. We should start to rely on intrinsic delivery of value — by being scientific, service‐oriented, and systems‐based practitioners — as the fundamental assurance of our survival and success. We should keep employing scientific principles and established methods of risk assessment to arrive at rigorous conclusions about what quality and safety really mean. We should keep working to ensure that regulatory and accreditation standards follow from these conclusions, rather than arbitrarily defining us and our work.

Inasmuch as no two medical imaging physicists are exactly alike, each will find unique answers to how we can best work scientifically, be service‐oriented, operate as mindful and effective contributors to the healthcare ecosystem, develop and use leadership traits and skills, and raise the profile of medical physics. It is imperative that we ask ourselves these questions now and continue to revisit them often. In doing so, we will recognize key practices that we need to sustain, and we will discover new pursuits that we need to adopt. Practically speaking, we will need to reduce or eliminate some current commitments of our time, energy, and attention to make room for the new and to reinforce those which we need to more deliberately sustain. These changes will provoke discomfort and we will tend to resist them. We will succeed by internalizing these elements, so that they become the foundation of the culture of our profession.

An integrated narrative of mission, vision, and value proposition for medical imaging physics, modeled on the HP Way, might look something like this:

We are scientists. We evaluate, recommend, and decide using data, collected and analyzed using sound, transparent, reproducible methods.
We are service‐oriented. We take initiative to discover and understand problems that inhibit our colleagues’ success and our patients’ health. We solve problems in a way that satisfies and delights our clients.
We are systems‐based. We strive to understand and consider the impact of our work on the individuals, organizations, and processes that make up the healthcare enterprise.
We are leaders. We build strong relationships with people and deliberately practice and improve interpersonal skills. We communicate effectively and we take action.
We are visible. We are engaged in the delivery of healthcare, and our colleagues understand and value our contributions. We are proud of who we are and what we do, and we share that pride with conviction.
We are medical physicists, and we improve the health of every patient, every day.

## Conflict of Interest

The authors have no conflicts to disclose related to the subject matter of this Editorial.
